# Fatal atypical reversible posterior leukoencephalopathy syndrome: a case report

**DOI:** 10.1186/1752-1947-7-14

**Published:** 2013-01-10

**Authors:** Stefanie Kristin Golombeck, Carsten Wessig, Camelia-Maria Monoranu, Ansgar Schütz, Laszlo Solymosi, Nico Melzer, Christoph Kleinschnitz

**Affiliations:** 1 Department of Neurology, University of Wuerzburg, Josef-Schneider-Straße 11, Wuerzburg, 97080, Germany; 2 Institute of Neuropathology, University of Wuerzburg, Josef-Schneider-Straße 2, Wuerzburg, 97080, Germany; 3 Department of Neuroradiology, University of Wuerzburg, Josef-Schneider-Straße 11, Wuerzburg, 97080, Germany; 4 Current address: Department of Neurology, University of Muenster, Albert-Schweitzer-Campus 1, Muenster, 48149, Germany

**Keywords:** Blood pressure, Cerebral autoregulation, Generalized cerebral edema, Reversible posterior leukoencephalopathy syndrome

## Abstract

**Introduction:**

Reversible posterior leukoencephalopathy syndrome – a reversible subacute global encephalopathy clinically presenting with headache, altered mental status, visual symptoms such as hemianopsia or cortical blindness, motor symptoms, and focal or generalized seizures – is characterized by a subcortical vasogenic edema symmetrically affecting posterior brain regions. Complete reversibility of both clinical signs and magnetic resonance imaging lesions is regarded as a defining feature of reversible posterior leukoencephalopathy syndrome. Reversible posterior leukoencephalopathy syndrome is almost exclusively seen in the setting of a predisposing clinical condition, such as pre-eclampsia, systemic infections, sepsis and shock, certain autoimmune diseases, various malignancies and cytotoxic chemotherapy, transplantation and concomitant immunosuppression (especially with calcineurin inhibitors) as well as episodes of abrupt hypertension. We describe for the first time clinical, radiological and histological findings in a case of reversible posterior leukoencephalopathy syndrome with an irreversible and fatal outcome occurring in the absence of any of the known predisposing clinical conditions except for a hypertensive episode.

**Case presentation:**

A 58-year-old Caucasian woman presented with a two-week history of subacute and progressive occipital headache, blurred vision and imbalance of gait and with no evidence for raised arterial blood pressure during the two weeks previous to admission. Her past medical history was unremarkable except for controlled arterial hypertension. Cerebral magnetic resonance imaging demonstrated cortical and subcortical lesions with combined vasogenic and cytotoxic edema atypical for both venous congestion and arterial infarction. Routine laboratory and cerebrospinal fluid parameters were normal. The diagnosis of reversible posterior leukoencephalopathy syndrome was established.

Within hours after admission the patient showed a rapidly decreasing level of consciousness, extension and flexion synergisms, bilaterally extensor plantar responses and rapid cardiopulmonary decompensation requiring ventilatory and cardiocirculatory support. Follow-up cerebral imaging demonstrated widespread and confluent cytotoxic edematous lesions in different arterial territories, global cerebral swelling, and subsequent upper and lower brainstem herniation. Four days after admission, the patient was declared dead because of brain death.

**Conclusion:**

This case demonstrates that fulminant and fatal reversible posterior leukoencephalopathy syndrome may occur spontaneously, that is, in the absence of any of the known predisposing systemic conditions.

## Introduction

In 1996 Hinchey *et al*. [1] described reversible posterior leukoencephalopathy syndrome (RPLS): a reversible subacute global encephalopathy clinically presenting with headache, altered mental status, visual symptoms (hemianopsia or cortical blindness), motor symptoms, and focal or generalized seizures [1-4]. The characteristic neuroimaging feature in classical RPLS is a partially or completely reversible subcortical vasogenic edema (leukoencephalopathy) symmetrically affecting the posterior (parietal and occipital) brain regions [1,2,4,5]. Magnetic resonance imaging (MRI) exhibits transient signal alterations indicative of vasogenic edema [6,7]. By contrast, persisting signal alterations indicating cytotoxic edema due to secondary infarction are uncommon initial findings in RPLS [2,5]. RPLS is almost exclusively seen in the setting of a predisposing clinical condition, such as pre-eclampsia, systemic infections, sepsis and shock, certain autoimmune diseases, various malignancies, chemotherapy, transplantation and concomitant immunosuppression (especially with calcineurin inhibitors) as well as episodes of abrupt hypertension [2,4].

## Case presentation

**Figure 1:**
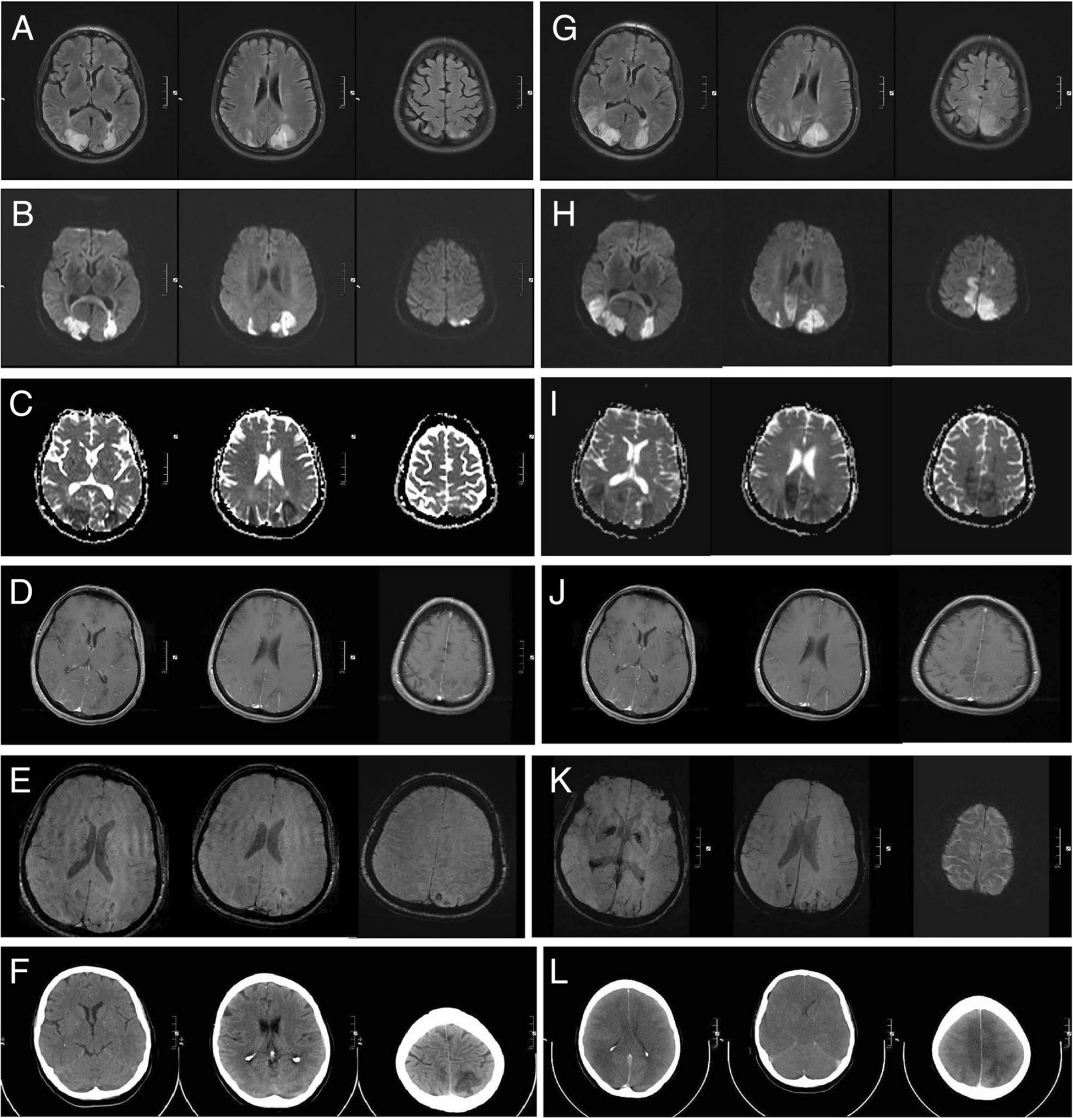
**Serial magnetic resonance imaging (3 Tesla) and computed tomography scans showing progressive bilateral reversible posterior leukoencephalopathy syndrome.** (**A–E**) Magnetic resonance imaging immediately after the patient was admitted showed (**A**) marked hyperintensity in T2 and fluid-attenuated inversion recovery (FLAIR) sequences of the posterior lesions. Diffusion-weighted imaging exhibited restricted diffusion (**B**) with a decreased signal on apparent diffusion coefficient mapping (**C**) consistent with cytotoxic edema. Lesions showed slight contrast enhancement (**D**). Susceptibility-weighted imaging (SWI) showed signal loss indicating the beginning of hemorrhagic lesion transformation (**E**). (**F–J**) Magnetic resonance imaging two days after the patient was admitted showed progressive confluent lesions in T2 and FLAIR sequences (**F**). Increasing areas of restricted diffusion (**G**) and volume expansion (**H**) indicated progressive cytotoxic edema. Lesions were still slightly contrast enhancing (**I**). SWI showed progressive signal loss indicating advancing hemorrhagic lesion transformation (**J**). (**K**) A computed tomography scan immediately after admission demonstrates well-demarcated bilateral hypodensities in posterior brain regions. (**L**) The computed tomography scan on day 3 after admission reveals global cerebral swelling, subsequent upper and lower brainstem herniation and brainstem compression as well as generalized thrombosis of the cerebral sinus and veins.

A 58-year-old Caucasian woman presented with a two-week history of subacute and progressive occipital headache, blurred vision and imbalance of gait and with no evidence available supporting the notion of a raised arterial blood pressure during the two weeks previous to admission. Her past medical history was unremarkable except for arterial hypertension, and there was no family history of neurological or medical disease. Neurological examination on admission was normal. However, an initial cerebral computed tomography (CT) scan showed bilateral posterior hypodense lesions (Figure 1K). An MRI of the brain on the same day demonstrated cortical and subcortical lesions in the occipital lobes with combined vasogenic and cytotoxic edema atypical for both venous congestion and arterial infarction (Figure 1A–D). Combined arterial and venous MR-angiography was normal ruling out thrombosis of cerebral sinus or veins and arterial thromboembolism as underlying causes. The diagnosis of somewhat atypical advanced RPLS was made.

Except for initial arterial hypertension (mean arterial blood pressure of about 130mmHg) the mean arterial blood pressure was kept well below 110mmHg by antihypertensive therapy. Cerebral vascular ultrasound, echocardiographic examination and continuous electrocardiography did not show any abnormalities. Routine laboratory parameters and parameters indicating a hypercoagulable state or vasculitis were normal as was routine cerebrospinal fluid analysis.

Within hours after admission the patient showed a rapidly decreasing level of consciousness, extension and flexion synergisms, bilaterally extensor plantar responses and rapid cardiopulmonary decompensation requiring ventilatory and cardiocirculatory support. Follow-up MRI two days after admission demonstrated more widespread and confluent cytotoxic edematous lesions in different arterial territories clearly exceeding the vertebrobasilar territory (Figure 1F–H), hemorrhagic transformation (Figure 1J) and mild contrast enhancement (Figure 1I). Of importance, at this time there was no evidence of thrombosis of the cerebral sinus and veins or arterial occlusion.

By contrast, three days after admission follow-up CT revealed global cerebral swelling, subsequent upper and lower brainstem herniation and brainstem compression as well as generalized thrombosis of the cerebral sinus and veins (Figure 1L). Four days after admission, the patient was declared dead because of brain death.

**Figure 2:**
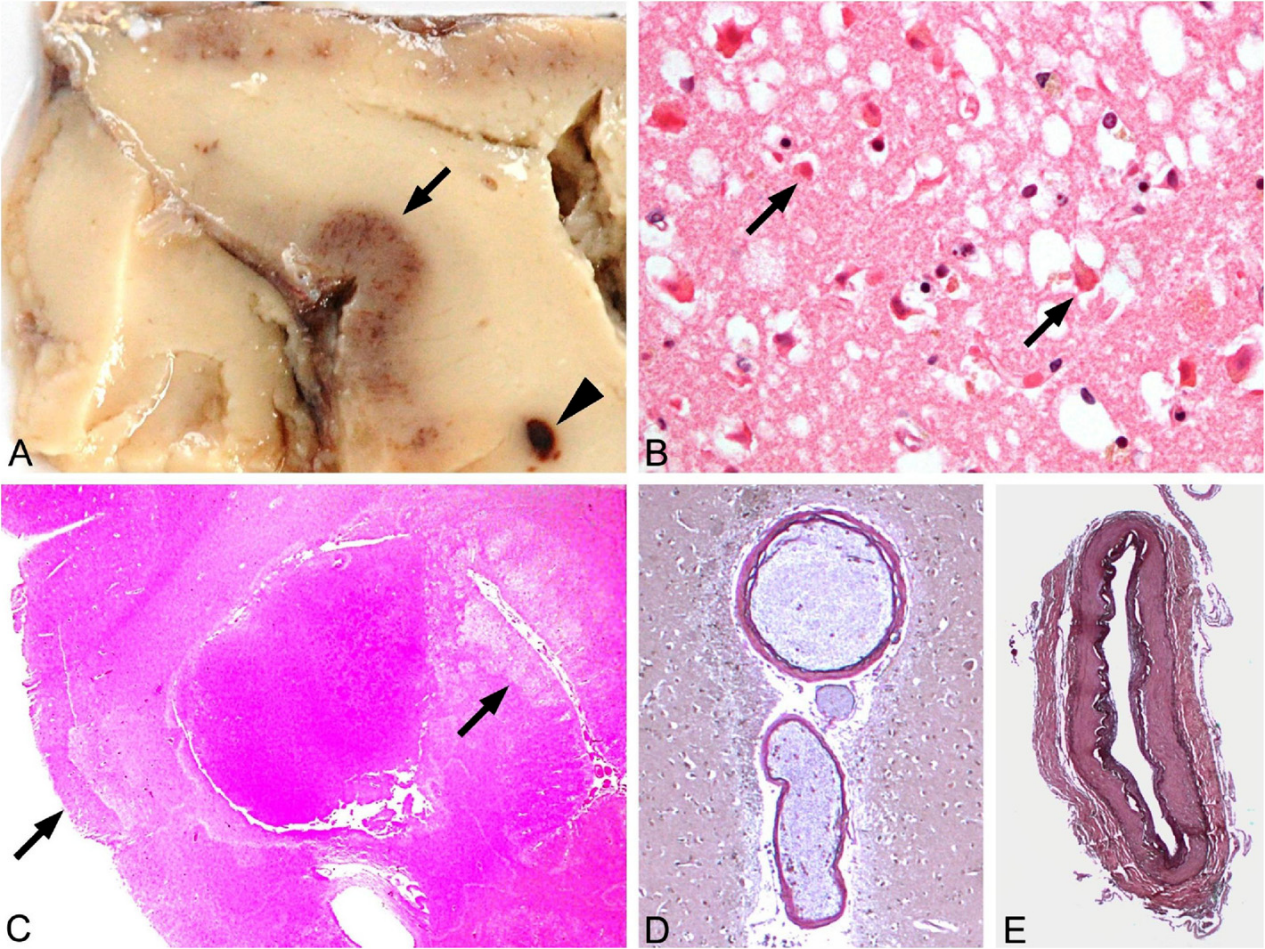
**Neuropathological analysis revealing laminar necrosis and hypoxic neuronal cell death in the absence of overt arterial pathology.** (**A,B**) Gross appearance revealed cortical discoloration due to laminar necrosis (→) and small white matter hemorrhage (►) in occipitotemporal regions. (**C**) Histopathological analysis showed hypoxic neuronal damage (arrows) (that is, nerve cells with eosinophilic and shrunken cytoplasm and hyperchromatic condensed nucleus (hematoxylin and eosin stain ×100)) (**D,E**) but no evidence of any arterial vascular pathology except for minor arteriosclerosis of intracerebral (**D**) and basal vessels (**E**) (elastica-van Gieson stain ×100).

Gross neuropathological analysis revealed generalized cerebral edema, consecutive upper and lower herniation with brain stem compression and concomitant general thrombosis of the cerebral sinus and veins. Of importance, there was no evidence of any additional arterial or venous pathology. Histological evaluation showed isolated hypoxic neuronal damage preferentially in the basal ganglia, hippocampus, pons, medulla oblongata and substantia nigra and laminar necrosis with edema in several cortical areas (Figure 2A–C). Of importance, there was no evidence of occult cancer or any another impairment of major organ systems at general autopsy.

## Discussion

Controversy exists about the mechanism underlying RPLS: (1) severe hypertension exceeding the upper limit of cerebral vascular autoregulation leading to reversible cerebral vasogenic edema [2,5,8] or (2) mild-to-moderate hypertension in the presence of a predisposing systemic condition leading to enhanced cerebral autoregulatory vasoconstriction and subsequent brain edema [8].

In our case, none of the predisposing systemic conditions were present and hypertension never exceeded the upper limit of cerebral vascular autoregulation (corresponding to a mean arterial pressure of 150–160mmHg in normotensive individuals), especially as there was chronic arterial hypertension, known to shift the upper limit of autoregulation of cerebral blood flow towards higher values [2,5,8]. Moreover, as the initially affected brain areas are not drained by deep cerebral veins, occult sinus vein thrombosis seems unlikely as an initiating event. Further, formation of widespread confluent cytotoxic edematous lesions occurred well before secondary thrombosis of cerebral sinus and veins became evident.

## Conclusion

Our case demonstrates that fulminant and fatal RPLS may occur spontaneously, that is, in the absence of any of the known predisposing systemic conditions.

## Consent

Written informed consent was obtained from the patient’s next of kin for publication of this case report and accompanying images. A copy of the written consent is available for review by the Editor-in-Chief of this journal.

## Abbreviations

CT: Computed tomography; FLAIR: fluid-attenuated inversion recovery; RPLS: Reversible posterior leukoencephalopathy syndrome; SWI: Susceptibility-weighted imaging.

## Competing interests

The authors declare that they have no competing interests.

## Authors’ contributions

SKG and CW acquired clinical data, CMM performed and interpreted pathological examinations, AS and LS performed and interpreted neuroradiological examinations, and NM and CK wrote the manuscript. All authors read and approved the final manuscript.
